# Uncontrolled Diabetes as an Associated Factor with Dynapenia in Adults Aged 50 Years or Older: Sex Differences

**DOI:** 10.1093/gerona/glz257

**Published:** 2019-10-28

**Authors:** Clarice Cavalero Nebuloni, Roberta de Oliveira Máximo, Cesar de Oliveira, Tiago da Silva Alexandre

**Affiliations:** 1 Gerontology Graduate Program, Federal University of Sao Carlos, Brazil; 2 Geriatrics and Gerontology Sector, Federal University of Sao Paulo, Brazil; 3 Physical Therapy Graduate Program, Federal University of Sao Carlos, Sao Carlos, SP, Brazil; 4 Department of Epidemiology and Public Health, University College London, UK; 5 Gerontology Department, Federal University of Sao Carlos, Brazil

**Keywords:** Glycated hemoglobin, Hyperglycemia, Neuromuscular strength, Dynapenia, Aging

## Abstract

**Background:**

Epidemiological studies demonstrate an association between diabetes and low neuromuscular strength (NMS). However, none have grouped participants into nondiabetics (ND), undiagnosed diabetics (UDD), controlled diabetics (CD), and uncontrolled diabetics (UCD) or investigated what glycated hemoglobin levels (HbA1c) are associated with low NMS (dynapenia) by sex.

**Methods:**

We analyzed the association between UDD, CD, and UCD and dynapenia, the extent to which the different groupings of these individuals modifies this association and the association between HbA1c levels and NMS, by sex, in a cross-sectional study involving 5,290 participants ≥50 years from the ELSA study. In the first two analyses, logistic regression models were used with dynapenia (grip strength <26 kg in men and <16 kg in women) as outcome and diabetes (ND, UDD, CD, and UCD) as exposure. Next, linear regression was performed with grip strength as the outcome, and the participants were classified based on HbA1c level as exposure. The models were adjusted by sociodemographic, behavioral, and clinical characteristics.

**Results:**

Compared to ND, only UCD was associated with dynapenia (men OR = 2.37 95% CI 1.36–4.14; women OR = 1.67 95% CI 1.01–2.79). This association was less clear, particularly in women, when CD and UCD groups were merged. HbA1c ≥6.5% in men and ≥8.0% in women were associated with lower NMS.

**Conclusions:**

UCD increases the chance of dynapenia in both sexes. The different groupings based on diabetes status modify the association between UCD and dynapenia. The threshold of HbA1c associated with reduced NMS is lower in men compared to women.

The mechanisms by which the greater decline in neuromuscular strength (NMS) occurs in individuals with diabetes and the level of glycated hemoglobin (HbA1c) considered harmful to the maintenance of NMS in this population are not fully known.

Epidemiological studies offer conflicting results. A longitudinal investigation involving 1,840 participants found that both men and women with diabetes had a greater loss of strength in their lower limbs, but not the upper limbs (1). Another longitudinal study involving 984 participants with a broad age range demonstrated that NMS loss was greater in diabetic participants with HbA1c higher than 6.1% (2). On the other hand, in a cross-sectional study involving 269 men aged 65 years or older, Yoon and collaborators found no differences in NMS between diabetics and nondiabetics or between the different strata of HbA1c. In a subanalysis involving only those with diabetes, however, the authors found an association between the lowest quartiles of muscle quality and HbA1c ≥8.5% (3).

Prolonged hyperglycemia is considered the cause of harm to muscle and its innervations, with a change in muscle strength and function in individuals with diabetes due to the increase in reactive oxygen species, the glycation of proteins, and the formation of advanced glycation end-products, leading to macrovascular and microvascular damages (4). Furthermore, chronic hyperglycemia increases the basal production of hepatic glucose and induces insulin resistance in the liver and skeletal muscle, which can exert a negative impact on NMS (5).

However, epidemiological studies analyzing the association between diabetes and low NMS (dynapenia) have neither considered the high prevalence of undiagnosed diabetics nor separated individuals in controlled and uncontrolled diabetes, which is an important factor to consider due to prolonged hyperglycemia exposure. In addition, the current evidence has not investigated sex differences. Men have higher NMS and also a greater NMS loss rate over time compared to women (6). Therefore, four hypotheses were tested in the present study: (a) there is an association between dynapenia and uncontrolled diabetes; (b) the different groupings of individuals according to their diabetes status modify the association between dynapenia and diabetes; (c) high HbA1c serum levels are associated with lower NMS in individuals aged 50 years and older; and (d) there are sex differences in these associations.

## Methods

### Study Population

ELSA is an ongoing panel study of community-dwelling individuals aged 50 years and over in England that commenced in 2002. The ELSA sample was drawn from participants who had previously participated in the Health Survey for England (7). After baseline, follow-up interviews occur biannually and health examinations every 4 years. A detailed description of the study can be found elsewhere (8).

This cross-sectional study used data from wave 6 (2012–2013), which was composed of 9,169 individuals aged ≥50 and older, 7,730 of whom underwent blood exams. A total of 5,814 blood samples were adequate for analysis, and 524 individuals were excluded for having incomplete information regarding the control variables. Therefore, our analytic sample comprised 5,290 individuals.

### Assessment of Neuromuscular Strength

Grip strength (GS) was measured using a dynamometer (Smedley’s). The participant remained standing with the arm alongside the trunk and the elbow at 90°. Three trials were performed with each hand, with a 1-min rest between trials. The highest value for the dominant hand was used in our analysis (9). To test the first and second hypotheses, men with GS <26 kg and women with GS <16 kg were considered to have dynapenia (9–12). To test the third hypothesis, GS was considered a continuous variable.

### Diabetes

Diabetes was defined by a self-report and confirmed by HbA1c. To test the first two hypotheses, the participants were classified into four groups: nondiabetic (ND)—no self-reported diabetes and HbA1c <5.7% or 5.7% to <6.5%; undiagnosed diabetics (UDD)—no self-reported diabetes combined with HbA1c ≥6.5%; controlled diabetic (CD)—self-reported diabetes, and HbA1c <7%; and uncontrolled diabetic (UCD)—self-reported diabetes and HbA1c ≥7%. To test the third hypothesis, the participants were classified according to HbA1c level: <6.5%; 6.5 to <7.0%; 7.0 to <8.0%; and ≥8.0% (13).

### Covariates

The sociodemographic variables were age group (50–59, 60–69, 70–79, 80–89, and ≥90), marital status (with or without conjugal life), level of education (0–11, 12–13, and >13 years of study), and household wealth (quintiles).

The behavioral characteristics were smoking (nonsmoker, ex-smoker, or smoker), frequency of alcohol intake (never/rarely, once a week, two to six times a week, daily, or no answer), and physical activity (PA). Self-reported PA data were collected using three questions on the frequency of participation in vigorous-, moderate-, and mild-intensity PA, with the response options for each being more than once per week, once per week, one to three times per month, or hardly ever. PA was further categorized into the following two groups: sedentary lifestyle (no activity on a weekly basis) or active (mild, moderate, or vigorous activity at least once a week) (11,14).

The health conditions were self-reported osteoarthritis, hypertension, cardiovascular disease, lung disease, osteoporosis, cancer, stroke, dementia, falls, hip fracture, and use of corticoids (systemic use, nasal preparations, and topic use). Depressive symptoms were defined by a score of ≥4 points on the Center for Epidemiologic Studies Depression Scale (15). Body mass index (BMI) was classified as ideal (18.5–24.9 kg/m^2^), underweight (<18.5 kg/m^2^), overweight (25.0–29.9 kg/m^2^), or obesity (≥30 kg/m^2^) (16). Waist circumference (WC) in centimeters was determined using a flexible metric tape at the midpoint between the last rib and upper margin of the iliac crest. For such, the participant remained standing with the arms alongside body with trunk free of clothing. The measurement was made with the abdomen relaxed at the end of expiration. Abdominal obesity was defined as a WC >102 cm for men and >88 cm for women (17,18).

### Statistical Analysis

The sample characteristics were expressed as means and proportion values. Differences between (1): men and women (2), the different diabetic groups, and (3) included and excluded individuals (due to missing data) were performed using the chi-square test and analysis of variance with Tukey’s post hoc test. Four logistic regression models for men and women separately were performed to test the first, second, and fourth hypotheses, using dynapenia as the outcome, since significant differences in NMS were found between men and women (*p* < .001).

In Model 1, the four diabetes status groups were analyzed separately: ND, UDD, CD, and UCD. From Models 2 to 4, different group combinations were used: Model 2 combined ND with UDD; Model 3 combined CD with UCD, and Model 4 combined ND with UDD and CD with UCD. Another three extra models with different combinations of diabetes status groups and their association with dynapenia are presented as supplementary material (Supplementary Table 5).

The odds ratios (ORs) of the models were used to calculate the percentage variation in the association between diabetes and dynapenia when the participants with diabetes were grouped inadequately. The percentage variation was calculated using the equation (*OR*_DM1_ – *OR*_DM*_)/*OR*_DM1,_ in which *OR*_DM1_ is the odd ratio of diabetes in Model 1 and *OR*_DM*_ is the odds ratio of diabetes in Models 2 to 4 and Supplementary Models.

The linear regression model, by sex, was used to test the third and fourth hypotheses. Univariate analyses were first performed in both the logistic and linear models. Control variables with a *p*-value of <.20 were incorporated into the multiple models using the *stepwise forward* method. The control variables included in the multivariate models were different for men and women. In the final model, a *p*-value of ≤.05 was considered indicative of a statistically significant association. The Stata 14 statistical package was used for all analyses.

### Ethical Approval and Informed Consent

The National Research Ethics Service (London Multicentre Research Ethics Committee [MREC/01/2/91]) approved the ELSA study. All participants gave written informed consent.

## Results

The mean age was 67 years, and the prevalence rates of UDD, CD, and UCD for men and women were 3.0 (95% CI: 2.3–3.7), 6.1 (95% CI: 5.2–7.1), 5.4 (95% CI: 4.6–6.4), 3.2 (95% CI: 2.6–3.9), 4.5 (95% CI: 3.8–5.4), and 3.8 (95% CI: 3.2–4.6), respectively. Mean GS was 39.3 kg for men and 23.5 kg for women, whereas the prevalence rates of dynapenia were 8.3% for men and 12.2% for women. The comparison of sociodemographic, behavioral, and clinical characteristics between men and women are shown in Tables 1 and 2.

**Table 1.** Sociodemographic and Behavioral Characteristics of 2,406 Men and 2,884 Women Aged ≥50 and Older, ELSA (2012–2013)

	Total *n* = 5,290	Men *n* = 2,406	Women *n* = 2,884
Age, years (*SD*)	66.6(8.9)	66.5(8.8)	66.7(8.9)
Age, %			
50–59	23.6	24.0	23.3
60–69	41.2	41.6	40.8
70–79	26.5	25.8	27.0
80–89	8.0	8.0	8.1
90 or older	0.7	0.6	0.8
Marital status (with conjugal life), %	67.5	75.2*	61.1*
Education, %			
>Level A	32.9	40.7*	26.5*
Level O or equivalent	29.0	26.0*	31.5*
<Level O or equivalent	38.1	33.3*	42.0*
Family wealth, %			
5th quintile (highest)	23.2	25.0*	21.8*
4th quintile	22.4	23.7*	21.4*
3rd quintile	21.4	21.5*	21.3*
2nd quintile	18.7	17.1*	19.9*
1st quintile (lowest)	14.3	12.7*	15.6*
Smoking, %			
Never smoked	38.2	32.6*	42.9*
Ex-smoker	50.5	56.3*	45.7*
Current smoker	11.3	11.1*	11.4*
Alcohol intake, %			
Never/rarely	18.7	12.2*	24.1*
Often	40.0	38.1*	41.5*
Daily	33.6	41.4*	27.1*
Did not answer	7.7	8.3*	7.3*
Physical activity, %			
Sedentary lifestyle	3.5	3.3	3.6

*Note:* Data expressed as percentage, mean, and standard deviation (*SD*) values. *Difference between sexes (*p* ≤.05).

**Table 2.** Clinical Characteristics of 2,406 Men and 2,884 Women Aged ≥50 and Older, ELSA (2012–2013)

	Total *n* = 5,290	Men *n* = 2,406	Women *n* = 2,884
Diabetes, %			
Nondiabetic (ND)	87.1	85.5*	88.5*
Undiagnosed diabetic (UDD)	3.1	3.0*	3.2*
Controlled diabetic (CD)	5.3	6.1*	4.5*
Uncontrolled diabetic (UCD)	4.5	5.4*	3.8*
HbA1c %, (SD)	5.9(0.8)	5.9(0.8)	5.9(0.7)
<6.5	90.1	88.9	90.9
6.5 to <7.0	4.5	4.8	4.3
7.0 to <8.0	2.9	3.4	2.5
≥8.0	2.5	2.9	2.3
Hypertension (yes), %	37.5	39.6*	35.8*
Cardiovascular disease (yes), %	15.7	17.3*	14.3*
Lung disease (yes), %	13.7	12.3*	14.8*
Osteoarthritis (yes), %	38.4	30.6*	44.9*
Osteoporosis (yes), %	7.9	3.0*	12.1*
Cancer (yes), %	5.0	5.4	4.7
Stroke (yes), %	3.5	3.9	3.2
Depression (yes), %	11.2	8.6*	13.5*
Dementia (yes), %	0.6	0.9*	0.4*
Falls (yes), %	20.2	17.8*	22.2*
Hip fracture (yes), %	0.3	0.4	0.3
Use of corticoids (yes), %	11.7	11.1	12.2
Waist circumference, cm (SD)	95.9(18.3)	101.6(21.8)*	91.2(13.2)*
>102 cm for men >88 cm for women (yes), %	50.6	43.5*	56.6*
Grip strength, kg (SD)	30.6(11.3)	39.3(9.6)*	23.5(6.6)*
<26 kg for men and <16 kg for women (yes), %	10.4	8.3*	12.2*
BMI, kg/m^2^ (SD)	28.0(5.0)	28.0(4.3)	28.0(5.5)
Underweight, %	0.9	0.4*	1.3*
Ideal, %	27.1	22.9*	30.7*
Overweight, %	42.5	49.3*	36.8*
Obesity, %	29.5	27.4*	31.2*

*Note:* Data expressed as percentage, mean, and standard deviation (SD) values. *Difference between sexes (*p* ≤.05).

The comparison between individuals included and those excluded due to missing data on diabetes, HbA1c, GS or covariates showed that excluded participants were older, had lower levels of education and wealth, lower alcohol intake, and were more sedentary. They also took fewer corticoids, had lower GS, greater WC and BMI, as well as greater prevalence rates of dynapenia, arterial hypertension, heart disease, cancer, stroke, depression, dementia, and hip fracture (Supplementary Table 1).

UCD, CD, and UDD men and women were older than the ND group. The women in the UCD, CD, and UDD groups had lower levels of education and wealth than those from the ND group. However the UCD women were wealthier than CD and UDD groups. Alcohol intake was lower for women in the UCD group than the ND, UDD, and CD groups and lower in the UCD group than the ND and CD groups in men. The UCD group had a higher mean HbA1c value compared to the other three groups in both sexes. The UDD men used more corticoids compared to the ND. Men and women in the ND group had a smaller WC and BMI and lower prevalence of hypertension and abdominal obesity. Men in the UCD, CD, and UDD groups had lower GS compared to the ND group. Among women, GS in CD and UCD groups was lower than in UDD and ND groups (Supplementary Tables 2 and 3).

Table 3 summarizes the results of the association between diabetes and dynapenia. According to Model 1 that classifies diabetes status into four groups, only those individuals in the UCD group shown more chance of dynapenia than ND (see results for covariates in Supplementary Table 4). Models 1 and 2 show that ND or ND+UDD as the reference group does not make much difference to the OR of having dynapenia for CD and UCD in men and women. Models 3 and 4 highlight that combining CD and UCD may mask the association between diabetes and dynapenia in women. Only the UCD group had a greater OR of dynapenia in men and women.

**Table 3.** Adjusted Logistic Regression Models for Chance of Dynapenia and Variation in Odds Ratio (*OR*) According to Different Groups of Diabetes Classification in Men (*n* = 2,406) and Women (*n* = 2,884) Aged ≥50 and Older, ELSA (2012–2013)

Models	Men*		Women^†^	
	Dynapenia *OR* (95% CI) *n* = 2,406	Percentage variation compared to Model 1 (%)	Dynapenia OR (95% CI) *n* = 2,884	Percentage variation compared to Model 1(%)
**Model 1**				
ND	1.00		1.00	
UDD	0.83 (0.35–1.98)		0.43 (0.18–1.02)	
CD	1.67 (0.95–2.94)		1.11 (0.68–1.83)	
UCD	2.37 (1.36–4.14)		1.67 (1.01–2.79)	
**Model 2**				
ND+UDD	1.00		1.00	
CD	1.69 (0.96–2.96)	+1.20	1.14 (0.69–1.88)	+2.7
UCD	2.40 (1.38–4.18)	+1.27	1.72 (1.03–2.87)	+3.0
**Model 3**				
ND	1.00		1.00	
UDD	0.83 (0.35–1.97)	−50.3	0.43 (0.18–1.02)	−61.3
CD+UCD	1.98 (1.30–3.02)	−16.5	1.35 (0.93–1.95)	−19.2
**Model 4**				
ND+UDD	1.00		1.00	
CD+UCD	2.00 (1.32–3.05)	−15.6	1.38 (0.96–2.00)	−17.4

*Note:* CI = Confidence interval; ND = Nondiabetic; UDD = Undiagnosed diabetic; CD = Controlled diabetic; UCD = Uncontrolled diabetic.

*Models for men were controlled by age, marital status, family wealth, lung disease, osteoarthritis, osteoporosis, depression, falls, and BMI.

^†^Models for women were controlled by age, marital status, education level, osteoarthritis, falls, and use of corticoids.

Table 4 displays the final multiple linear regression models with the results of the association between HbA1c level and GS. Men with HbA1c ≥6.5 and <7.0%; ≥7.0 and <8.0% and ≥8.0% exhibited a mean reduction in GS of 1.62 kg, 3.73 kg e 2.05 kg, respectively. Women with HbA1c ≥8.0% exhibited a mean reduction in GS of 1.77 kg (see results for all covariates on Supplementary Table 6).

**Table 4.** Association Between HbA1c and GS in 2,406 Men,and 2,884 Women Aged ≥50, and Older, ELSA (2012–2013)

HbA1c%	Multiple Linear Regression Model	
	*β* coefficient (95% CI)	
	Men^*^	Women^†^
<6.5	1.00	1.00
≥6.5 to <7.0	−1.62 (−3.19 to −0.04)	0.15 (−0.91 to 1.21)
≥7.0 to <8.0	−3.73 (−5.56 to −1.90)	−0.51 (−1.88 to 0.86)
≥8.0	−2.05 (−4.06 to −0.04)	−1.77 (−3.21 to −0.33)

*Note*: *Men’s model were controlled by age, stroke, osteoporosis, osteoarthritis, cancer, falls, depression, BMI, marital status, level of education, and dementia.

^†^Women’s model were controlled by age, dementia, stroke, osteoarthritis, level of education, use of corticoids, osteoporosis, depression, falls, and abdominal obesity.

## Discussion

The main findings of the present study are that men and women with uncontrolled diabetes have a greater chance of having dynapenia than those with ND. Distinguishing people with well-controlled versus not well-controlled diabetes modifies the association between UCD and dynapenia in epidemiological studies. Moreover, men with HbA1c ≥6.5% and women with HbA1c ≥8.0% have a significant reduction in GS.

The few studies that have analyzed the association between low NMS, diabetes, and high HbA1c levels report conflicting results. In a cross-sectional study involving 2,618 individuals aged 70–79 years, Park and coworkers (19). found that the only men with diabetes had less upper and lower limb strength compared to nondiabetic men. However, in a longitudinal study with a three-year follow up involving 1,840 participants, the same authors found that both men and women with diabetes had greater loss of knee extensor strength, but not upper limb strength (1). In a longitudinal study involving 984 participants 26 to 96 years of age, Kalyani and coworkers (2). found that the loss of strength was greater among individuals with the highest HbA1c levels. However, the authors categorized HbA1c in quartiles, with the highest quartile HbA1c ≥6.1%, which is considered a low value.

On the other hand, Yoon and coworkers (3). conducted a cross-sectional study and found no differences in NMS of the lower limbs among 269 men ≥65 years with and without diabetes or when considering different HbA1c strata (<6.5%, 6.5 to <7.5%, 7.5 to <8.5% and ≥8.5%). Besides strength, muscle mass, and quality were also evaluated in diabetic individuals. The authors only found an association between the lowest quartiles of muscle quality and HbA1c ≥8.5%. It should be stressed that the nonstandardization of cutoff points for the definition of dynapenia and the inadequate separation of diabetic groups may be important factors to the absence of such associations. Furthermore, such findings suggest that only high HbA1c levels are associated with poor muscle quality, which could be a misleading assumption.

However, no previous study has analyzed ND, UDD, CD, and UCD groups separately. Therefore, besides identifying that UCD was the only condition associated with dynapenia in men and women, the adoption of glycated hemoglobin categories based on the glycemic cutoff levels recommended by the American Diabetes Association (ADA), allowed us to identify that in men a decline in grip strength occurs with a HbA1c ≥6.5% and, in women, only with higher levels (≥8.0%) (13).

Despite the divergent results in previous studies, the greater occurrence of dynapenia and lower muscle strength in individuals with uncontrolled diabetes have been attributed to the effects of exposure to prolonged hyperglycemia (2,20–23).

High concentrations of blood sugar lead to peripheral nerve dysfunction due to demyelination and atrophy of the motor axon, affecting the capacity of nerve impulse conduction, reducing the regenerative potential (24) and causing the loss of nerve fibers (25). The main pathways responsible for these dysfunctions are the increased flow of the polyol pathway, the accelerated formation of advanced glycation end-products (AGEs), the activation of hexosamine and protein kinase C (26,27), and accentuated oxidative stress, which facilitate muscle atrophy and the loss of strength (27–29).

The cells that make up skeletal muscle also have several modifications due to exposure to prolonged hyperglycemia. There is evidence that fast-twitch fibers are more sensitive to the loss of strength when exposed to this condition (30). Exposure to prolonged hyperglycemia increases protein glycation in the muscle, favoring muscle atrophy, interruptions, and modifications in the structure of Z lines as well as morphological abnormalities in the skeletal muscle mitochondria, which hinders the production of energy for contractions and the generation of force (30).

The buildup of AGEs is regarded as a factor associated with lower NMS (31,32). In individuals with diabetes, this accumulation is accelerated, i.e., the higher the glycemia, the greater the formation of these components (33). In a cross-sectional study involving 36 Japanese patients with diabetes, Mori and coworkers (34). found that the buildup of AGEs was correlated with lower knee extensor strength, but not with sarcopenia. These findings lend support to the notion that the reduction in muscle strength and mass may not be linear and the reduction in strength may be mediated by mechanisms other than only the reduction in muscle mass in this population.

Additionally, there is evidence that diabetes exerts a negative effect on the quantity and/or function of satellite cells. While the mechanism for this dysfunction is not yet fully clarified, in vitro studies have demonstrated that satellite cells in a hyperglycemic medium have an increased predisposition to differentiate into adipocytes, which would negatively impact the quantity of contractile tissue in muscle (35,36).

This evidence of impairment to skeletal muscle and the peripheral nervous system, which culminate in compromised NMS, further underscores the fact that we found no association between dynapenia and controlled diabetes in the present study. This likely occurred because, although diabetics, HbA1c levels in men and women groups were indicative of good glycemic control, reducing the chance of muscular and neural harm caused by exposure to prolonged hyperglycemia, which underscores the importance of blood sugar control to the maintenance of NMS.

However, the gender differences found in the present study in the associations between HbA1c and GS could be explained. Women have physiologically a smaller NMS rate of loss, mass and muscular quality compared to men. In addition, women are more sensitive to insulin, have higher capillary density, and more fibers type I that are responsible for the oxidative metabolism of glucose and fatty acids (6,37,38). Therefore, they may utilize glucose more efficiently and therefore have their NMS affected at higher HbA1c levels than men. On the other hand, in men a greater damage to the muscular structure occurs due to a reduction in number and size of fibers type II (39) accompanied by reductions in both growth hormone and testosterone levels (40). In addition, there is evidence that men are less sensitive to insulin and have more visceral and hepatic fat (41) and, consequently, more susceptible to reductions in NMS at lower HbA1c levels than women.

The present study has strong points that should be considered. First, the study was conducted with a large sample of community-dwelling men and women aged 50 years and older, which enabled adequately dividing them into four groups by sex and diabetes status. Second, the different regression models enabled more assertive conclusions with regard to the association between uncontrolled diabetes and dynapenia. Third, the use of grip strength as an assessment method for NMS enabled the identification of the association between low upper limb strength and both uncontrolled diabetes and different HbA1c categories, as most previous studies have only found an association with lower limb strength.

This study also has limitations that need to be recognized. The study design does not enable the establishment of associations of causality or the extent to which survivorship bias may have exerted an influence on the associations encountered. Moreover, the individuals excluded from the sample for diverse reasons where older and had lower grip strength, a higher prevalence of dynapenia as well as generally worse socioeconomic and clinical status, which may have introduced some degree of bias into the results. However, despite the differences between the included and excluded individuals, it was possible to find associations between UCD and dynapenia as well as between HbA1c and low neuromuscular strength in men and women. Another limitation was the lack of information on the time since the diagnosis of diabetes and the nonevaluation of possible neuropathies.

In summary, individuals with UCD have a greater chance of exhibiting dynapenia than those with ND. Distinguishing people with well controlled versus not well-controlled diabetes modifies the association between UCD and dynapenia. Men and women with HbA1c ≥6.5% and ≥8.0%, respectively, have lower neuromuscular strength than those with HbA1c <6.5%. NMS in men is more susceptible to hyperglycemia than in women. Follow-up studies using mixed models in order to estimates the trajectories of NMS according different status of HbA1c are needed to confirm whether hyperglycemia is a risk factor for the development of dynapenia and whether maintaining HbA1c levels <7.0% may be a protective factor that minimizes the loss of neuromuscular strength.

## Supplementary Material

glz257_suppl_Supplementary_Tables
