# Supplementary material for: Uncontrolled Diabetes as an Associated Factor with Dynapenia in Adults Aged 50 Years or Older: Sex Differences
Source: J Gerontol A Biol Sci Med Sci. 2019 Oct 28;75(6):1191–7. doi: 10.1093/gerona/glz257 (PMC7243578; doi:10.1093/gerona/glz257)
Supplement: glz257_suppl_Supplementary_Tables [file glz257_suppl_supplementary_tables.docx]

**Supplementary Table 1** - Comparison between included and excluded men and women aged ≥ 50 years old from ELSA, Wave 6 (2012 - 2013).

|  | Men | | Women | |
| --- | --- | --- | --- | --- |
|  | **Included**  **n=2,406** | **Excluded**  **n=1,667** | **Included**  **n=2,884** | **Excluded**  **n=2,212** |
| Sociodemographic characteristics |  |  |  |  |
| Age, (years) | 66.5^*^ | 69.1^*^ | 66.7^§^ | 69.6^§^ |
| Age, % |  |  |  |  |
| 50-59 | 24.0^*^ | 20.8^*^ | 23.3^§^ | 21.0^§^ |
| 60-69 | 41.6^*^ | 33.5^*^ | 40.8^§^ | 32.3^§^ |
| 70-79 | 25.8^*^ | 28.9^*^ | 27.0^§^ | 25.5^§^ |
| 80-89 | 8.0^*^ | 14.0^*^ | 8.0^§^ | 15.7^§^ |
| 90 or older | 0.6^*^ | 2.8^*^ | 0.8^§^ | 5.5^§^ |
| Marital status (with conjugal life),% | 75.2^*^ | 70.1^*^ | 61.1^§^ | 52.4^§^ |
| Education,% |  |  |  |  |
| >Level A | 40.7^*^ | 35.5^*^ | 26.5^§^ | 22.9^§^ |
| Level O or equivalent | 26.0^*^ | 22.1^*^ | 31.5^§^ | 25.9^§^ |
| <Level O or equivalent | 33.3^*^ | 42.5^*^ | 42.1^§^ | 51.2^§^ |
| Family wealth,% |  |  |  |  |
| 5^th^ quintile (highest) | 25.0^*^ | 21.0^*^ | 21.8^§^ | 17.9^§^ |
| 4^th^ quintile | 23.7^*^ | 20.1^*^ | 21.4^§^ | 18.3^§^ |
| 3^rd^ quintile | 21.5^*^ | 19.8^*^ | 21.3^§^ | 20.9^§^ |
| 2^nd^ quintile | 17.1^*^ | 19.6^*^ | 19.9^§^ | 20.6^§^ |
| 1^st^ quintile (lowest) | 12.7^*^ | 19.4^*^ | 15.6^§^ | 22.3^§^ |
| Behavioral characteristics |  |  |  |  |
| Smoking,% |  |  |  |  |
| Never smoked | 32.6^*^ | 26.6^*^ | 42.9 | 43.6 |
| Ex-smoker | 56.3^*^ | 59.7^*^ | 45.7 | 43.6 |
| Current smoker | 11.1^*^ | 13.7^*^ | 11.4 | 12.8 |
| Alcohol intake,% |  |  |  |  |
| Never/rarely | 12.2^*^ | 14.0^*^ | 24.1^§^ | 25.8^§^ |
| Often | 38.2^*^ | 27.3^*^ | 41.5^§^ | 31.2^§^ |
| Daily | 41.4^*^ | 31.9^*^ | 27.2^§^ | 18.9^§^ |
| Did not answer | 8.3^*^ | 26.8^*^ | 7.3^§^ | 24.2^§^ |
| Sedentary lifestyle,% | 3.3^*^ | 13.7^*^ | 3.6^§^ | 14.1^§^ |
| Clinical Conditions |  |  |  |  |
| HbA1c %, (SD) | 5.9 (0.8)^*^ | 6.1 (1.0)^*^ | 5.9 (0.7) | 5.9 (0.8) |
| Diabetes,% |  |  |  |  |
| Non-diabetic (ND) | 85.5^*^ | 77.6^*^ | 88.5 | 87.2 |
| Undiagnosed diabetic (UDD) | 3.0^*^ | 7.3^*^ | 3.2 | 3.6 |
| Controlled diabetic (CD) | 6.1 | 6.4 | 4.5 | 5.6 |
| Uncontrolled diabetic (UCD) | 5.4^*^ | 8.7^*^ | 3.8 | 3.6 |
| Hypertension (yes),% | 39.6^*^ | 46.1^*^ | 35.8^§^ | 43.0^§^ |
| Cardiovascular disease (yes),% | 17.3^*^ | 28.8^*^ | 14.3^§^ | 21.8^§^ |
| Lung disease (yes),% | 12.3^*^ | 14.8^*^ | 14.8 | 16.4 |
| Osteoarthritis (yes),% | 30.6 | 32.9 | 44.9 | 47.1 |
| Osteoporosis (yes),% | 3.0 | 2.7 | 12.1^§^ | 15.3^§^ |
| Cancer (yes),% | 5.4^*^ | 7.9^*^ | 4.7^§^ | 6.9^§^ |
| Stroke (yes),% | 3.9^*^ | 8.2^*^ | 3.2^§^ | 6.7^§^ |
| Depression (yes),% | 8.6^*^ | 12.9^*^ | 13.5^§^ | 20.3^§^ |
| Dementia (yes),% | 0.9^*^ | 2.3^*^ | 0.4^§^ | 3.1^§^ |
| Falls (yes),% | 17.8^*^ | 24.2^*^ | 22.2 | 24.8 |
| Hip fracture (yes),% | 0.4^*^ | 1.0^*^ | 0.3^§^ | 1.2^§^ |
| Use of corticoids (yes),% | 11.1^*^ | 8.1^*^ | 12.2^§^ | 9.9^§^ |
| Anthropometry and physical performance | | | | |
| Waist circumference, cm (SD) | 101.6 (21.8)^*^ | 103.9 (12.8)^*^ | 91.2 (13.2)^§^ | 94.2 (29.1)^§^ |
| Grip strength – kg (SD) | 39.3 (9.6)^*^ | 36.1 (10.4)^*^ | 23.5 (6.6)^§^ | 21.8 (7.4)^§^ |
| Dynapenia, (yes) % | 8.3^*^ | 15.4^*^ | 12.2^§^ | 19.8^§^ |
| BMI – kg/m^2^ (SD) | 28.0 (4.3)^*^ | 28.8 (5.0)^*^ | 28.0 (5.5)^§^ | 29.1 (6.4)^§^ |

Data expressed as percentage and mean values. ^*^Difference between men included and excluded (p value ≤0.05). ^§^Difference between women included and excluded (p value ≤0.05).

**Supplementary Table 2** – Sociodemographic, behavioral and clinic characteristics of 2,406 men aged ≥50 years old according diabetes status, ELSA (2012-2013).

|  | **Non-diabetic**  **n=2,057** | **Undiagnosed diabetic**  **n=71** | **Controlled diabetic**  **n=147** | **Uncontrolled diabetic**  **n=131** |
| --- | --- | --- | --- | --- |
| **Sociodemographic characteristics** |  |  |  |  |
| Age, years (SD) | 66.0 (8.8) | 70.7 (9.0)^a^ | 68.6 (8.0)^a^ | 68.7 (8.4)^a^ |
| Age, % |  |  |  |  |
| 50-59 | 25.6 | 11.3^a^ | 15.0^a^ | 15.3^a^ |
| 60- 69 | 41.8 | 36.6^a^ | 42.2^a^ | 39.6^a^ |
| 70-79 | 24.5 | 35.2^a^ | 33.3^a^ | 33.6^a^ |
| 80-89 | 7.4 | 16.9^a^ | 9.5^a^ | 10.7^a^ |
| 90 or older | 0.7 | 0.0 | 0.0 | 0.8 |
| Marital status (with conjugal life),% | 75.7 | 69.0 | 74.8 | 70.2 |
| Education,% |  |  |  |  |
| >Level A | 41.9 | 35.2 | 34.0 | 32.1 |
| Level O or equivalent | 25.8 | 25.4 | 27.2 | 28.2 |
| <Level O or equivalent | 32.3 | 39.4 | 38.8 | 39.7 |
| Family wealth,% |  |  |  |  |
| 5th quintile (highest) | 26.2 | 18.3^a^ | 24.5 | 9.8^a,c^ |
| 4th quintile | 24.7 | 14.1^a^ | 17.7 | 19.9^a,c^ |
| 3rd quintile | 21.5 | 21.1 | 21.7 | 21.4 |
| 2nd quintile | 16.0 | 26.8^a^ | 22.5 | 22.9^a^ |
| 1st quintile (lowest) | 11.6 | 19.7^a^ | 13.6 | 26.0^a,c^ |
| **Behavioral characteristics** |  |  |  |  |
| Smoking,% |  |  |  |  |
| Never smoked | 33.6 | 28.2 | 25.2 | 26.7 |
| Ex-smoker | 55.3 | 59.1 | 63.3 | 62.6 |
| Current smoker | 11.1 | 12.7 | 11.5 | 10.7 |
| Alcohol intake,% |  |  |  |  |
| Never/rarely | 10.6 | 22.5^a^ | 17.0 | 27.5^a,c^ |
| Often | 37.9 | 38.0 | 37.4 | 42.8^a,c^ |
| Daily | 43.5 | 29.6^a^ | 37.4 | 18.2^a,c^ |
| Did not answer | 8.0 | 9.9^a^ | 8.2 | 11.5^a,c^ |
| Physical activity,% |  |  |  |  |
| Sedentary lifestyle | 2.6 | 9.9^a^ | 6.1 | 6.9^a^ |
| **Clinical Conditions** |  |  |  |  |
| HbA1c %, (SD) | 5.7 (0.3) | 7.0 (0.8)^a^ | 6.3 (0.5)^a,b^ | 8.4 (1.3)^a,b,c^ |
| Hypertension (yes),% | 35.3 | 54.9^a^ | 67.4^a^ | 67.9^a^ |
| Cardiovascular disease (yes),% | 15.6 | 26.8^a^ | 27.9^a^ | 28.2^a^ |
| Lung disease (yes),% | 11.9 | 18.3 | 14.3 | 14.5 |
| Osteoarthritis (yes),% | 29.2 | 47.9^a^ | 36.1 | 38.2^a^ |
| Osteoporosis (yes),% | 2.8 | 4.2 | 2.7 | 5.3 |
| Cancer (yes), % | 5.3 | 5.6 | 7.5 | 5.3 |
| Stroke (yes),% | 3.0 | 9.9^a^ | 8.8^a^ | 9.2^a^ |
| Depression (yes),% | 7.9 | 11.3 | 12.2 | 13.0^a^ |
| Dementia (yes),% | 0.7 | 5.6^a^ | 0.0^b^ | 3.1^a,c^ |
| Falls (yes),% | 16.4 | 26.8^a^ | 24.5^a^ | 27.5^a^ |
| Hip fracture (yes),% | 0.4 | 0.0^a^ | 0.7^a^ | 0.0^a^ |
| Use of corticoids (yes),% | 10.4 | 22.5^a^ | 15.0 | 12.2 |
| **Anthropometry and physical performance** |  |  |  |  |
| Waist circumference, cm (SD) | 100.4 (22.7) | 110.9 (13.7)^a^ | 106.2 (12.7)^a,b^ | 110.9 (13.2)^a,c^ |
| >102cm for men >88cm for women (yes),% | 39.3 | 69.0^a^ | 63.3^a^ | 73.3^a^ |
| Grip strength – kg (SD) | 39.8 (9.5) | 36.0 (9.5)^a^ | 37.1 (9.9)^a^ | 35.4 (10.4)^a^ |
| <26kg for men and <16kg for women (yes),% | 7.1 | 11.3 | 13.6^a^ | 19.1^a^ |
| BMI – kg/m^2^ (SD) | 27.6 (4.0) | 31.0 (5.0)^a^ | 29.6 (5.2)^a^ | 31.0 (5.0)^a,c^ |
| Underweight,% | 0.3 | 0.0^a^ | 0.7^a^ | 0.8^a^ |
| Ideal,% | 24.7 | 12.7^a^ | 15.7^a^ | 9.8^a^ |
| Overweight,% | 51.3 | 32.4^a^ | 42.8^a^ | 34.4^a^ |
| Obesity,% | 23.7 | 54.9^a^ | 40.8^a^ | 55.0^a^ |

Data expressed as percentage, mean and standard deviation (SD) values. ^a^ Significantly different from non-diabetics. ^b^ Significant different from undiagnosed diabetics. ^c^ Significantly different from controlled diabetics. Significance was accepted between groups with p value ≤0.05.

**Supplementary Table 3** – Sociodemographic, behavioral and clinic characteristics of 2,884 women aged ≥50 years old according diabetes status, ELSA (2012-2013).

|  | Non-diabetic  n=2,552 | Undiagnosed diabetic  n=91 | Controlled diabetic  n=131 | Uncontrolled diabetic  n=110 |
| --- | --- | --- | --- | --- |
| **Socioeconomic characteristics** |  |  |  |  |
| Age, years (SD) | 66.4 (8.8) | 68.7 (8.6)^a^ | 70.8 (9.4)^a^ | 68.7 (9.2)^a^ |
| Age, % |  |  |  |  |
| 50-59 | 24.1 | 15.4^a^ | 14.5^a^ | 20.0 |
| 60-69 | 41.7 | 37.4^a^ | 28.2^a^ | 39.1 |
| 70-79 | 26.1 | 39.5^a^ | 35.9^a^ | 27.3 |
| 80-89 | 7.4 | 6.6^a^ | 19.9^a^ | 10.9 |
| 90 or older | 0.7 | 1.1^a^ | 1.5^a^ | 2.7 |
| Marital status (with conjugal life),% | 61.9 | 52.8^a^ | 58.0^a^ | 52.7 |
| Education,% |  |  |  |  |
| >Level A | 27.8 | 20.9^a^ | 17.6^a^ | 10.9^a^ |
| Level O or equivalent | 31.6 | 25.3^a^ | 30.5^a^ | 35.5^a^ |
| <Level O or equivalent | 40.6 | 53.8^a^ | 51.9^a^ | 53.6^a^ |
| Family wealth,% |  |  |  |  |
| 5th quintile (highest) | 23.3 | 16.5^a^ | 9.9^a^ | 21.8^a,b,c^ |
| 4th quintile | 21.9 | 16.5^a^ | 23.7^a^ | 21.4^a,b,c^ |
| 3rd quintile | 20.8 | 26.4^a^ | 19.1^a^ | 21.3^a,b,c^ |
| 2nd quintile | 20.1 | 17.6^a^ | 19.8^a^ | 19.9^a,b^ |
| 1st quintile (lowest) | 13.9 | 23.0^a^ | 27.5^a^ | 15.6^a,b,c^ |
| **Behavioral characteristics** |  |  |  |  |
| Smoking,% |  |  |  |  |
| Never smoked | 43.4 | 38.4 | 39.7 | 42.9 |
| Ex-smoker | 45.4 | 44.0 | 50.4 | 45.7 |
| Current smoker | 11.2 | 17.6 | 9.9 | 11.4 |
| Alcohol intake,% |  |  |  |  |
| Never/rarely | 22.1 | 29.7^a^ | 42.0^a^ | 44.5^a,b,c^ |
| Often | 42.0 | 44.0^a^ | 36.6^a^ | 33.6^a,b,c^ |
| Daily | 29.1 | 14.3^a^ | 15.3^a^ | 6.4^a,b,c^ |
| Did not answer | 6.8 | 12.0^a^ | 6.1^a^ | 15.5^a,b,c^ |
| Physical activity,% |  |  |  |  |
| Sedentary lifestyle | 2.9 | 3.3 | 9.9^a^ | 14.6^a,b^ |
| **Clinical Conditions** |  |  |  |  |
| HbA1c %, (SD) | 5.7 (0.3) | 7.1 (0.9)^a^ | 6.4 (0.4)^a,b^ | 8.4 (1.3)^a,b,c^ |
| Hypertension (yes),% | 32.0 | 60.4^a^ | 67.2^a^ | 66.4^a^ |
| Cardiovascular disease (yes),% | 13.0 | 16.5 | 28.2^a,b^ | 26.4^a^ |
| Lung disease (yes),% | 14.3 | 20.9 | 15.3 | 20.9 |
| Osteoarthritis (yes),% | 43.7 | 48.4 | 56.5^a^ | 56.4^a^ |
| Osteoporosis (yes),% | 11.7 | 11.0 | 17.6^a^ | 14.6 |
| Cancer (yes),% | 4.6 | 4.4 | 6.1 | 5.5 |
| Stroke (yes),% | 2.8 | 6.6^a^ | 3.8 | 9.1^a^ |
| Depression (yes),% | 12.2 | 14.3 | 26.0^a,b^ | 26.4^a,b^ |
| Dementia (yes),% | 0.4 | 0.0 | 0.0 | 1.8^a^ |
| Falls (yes),% | 21.4 | 25.3 | 30.5^a^ | 27.3 |
| Hip fracture (yes),% | 0.2 | 1.1^a^ | 0.8^a^ | 0.0 |
| Use of corticoids (yes),% | 11.8 | 16.5 | 14.5 | 14.6 |
| **Anthropometry and physical performance** |  |  |  |  |
| Waist circumference, cm (SD) | 89.9 (12.6) | 100.3 (11.1)^a^ | 100.6 (13.3)^a^ | 103.5 (13.0)^a^ |
| >102cm for men >88cm for women (yes),% | 52.8 | 86.8^a^ | 83.2^a^ | 88.2^a^ |
| Grip strength – kg (SD) | 23.7 (6.5) | 23.5 (5.9) | 21.1 (6.9)^a,b^ | 21.1 (7.3)^a,b^ |
| <26kg for men and <16kg for women (yes),% | 11.6 | 6.6 | 19.1^a,b^ | 22.7^a,b^ |
| BMI – kg/m^2^ (SD) | 27.6 (5.3) | 31.0 (5.6)^a^ | 31.4 (6.5)^a^ | 31.9 (6.1)^a^ |
| Underweight,% | 1.4 | 0.0^a^ | 0.0^a^ | 0.0^a^ |
| Ideal,% | 33.0 | 13.2^a^ | 14.5^a^ | 10.9^a^ |
| Overweight,% | 37.7 | 29.7^a^ | 29.0^a^ | 30.0^a^ |
| Obesity,% | 27.9 | 57.1^a^ | 56.5^a^ | 59.1^a^ |

Data expressed as percentage, mean and standard deviation (SD) values. ^a^ Significantly different from non-diabetics. ^b^ Significant different from undiagnosed diabetics. ^c^ Significantly different from controlled diabetics. Significance was accepted between groups with p value ≤0.05.

**Supplementary Table 4** – Final logistic regression models for dynapenia in men and women ≥50 years old, ELSA (2012-2013).

| **Men (n=2,406)** | **OR** | **95% CI** |
| --- | --- | --- |
| Diabetes |  |  |
| Non-diabetic | 1.00 |  |
| Undiagnosed diabetic | 0.83 | 0.35 – 1.98 |
| Controlled diabetic | 1.67 | 0.95 – 2.94 |
| Uncontrolled diabetic | 2.37 | 1.36 – 4.14 |
| Age, (years) |  |  |
| 50-59 | 1.00 |  |
| 60-69 | 2.60 | 1.27 – 5.33 |
| 70-79 | 7.86 | 3.91 – 15.8 |
| 80-89 | 27.1 | 12.9 – 57.0 |
| 90 or older | 65.9 | 18.5 – 235.1 |
| Marital status, (with conjugal life) | 1.54 | 1.07 – 2.20 |
| Family wealth |  |  |
| 5^th^ quintile (highest) | 1.00 |  |
| 4^th^ quintile | 1.02 | 0.59 – 1.77 |
| 3^rd^ quintile | 1.25 | 0.73 – 2.13 |
| 2^nd^ quintile | 1.71 | 0.99 – 2.94 |
| 1^st^ quintile (lowest) | 2.18 | 1.23 – 3.88 |
| Lung disease, (yes) | 1.53 | 1.01 – 2.36 |
| Osteoarthritis, (yes) | 1.65 | 1.17 – 2.31 |
| Osteoporosis, (yes) | 1.90 | 0.96 – 3.76 |
| Depression, (yes) | 3.36 | 2.10 – 5.38 |
| Falls, (yes) | 1.56 | 1.09 – 2.24 |
| BMI – kg/m^2^ |  |  |
| Ideal | 1.00 |  |
| Underweight | 2.36 | 0.30 – 18.33 |
| Overweight | 0.72 | 0.49 – 1.07 |
| Obesity | 0.46 | 0.28 – 0.75 |
| **Women (n=2,884)** | **OR** | **95% CI** |
| Diabetes |  |  |
| Non-diabetic | 1.00 |  |
| Undiagnosed diabetic | 0.43 | 0.18 – 1.02 |
| Controlled diabetic | 1.11 | 0.68 – 1.83 |
| Uncontrolled diabetic | 1.67 | 1.01 – 2.79 |
| Age, (years) |  |  |
| 50-59 | 1.00 |  |
| 60-69 | 0.90 | 0.61 – 1.34 |
| 70-79 | 1.51 | 1.02 – 2.24 |
| 80-89 | 5.29 | 3.38 – 8.27 |
| 90 or older | 13.49 | 5.39 – 33.81 |
| Marital status, (with conjugal life) | 1.31 | 1.02 – 1.69 |
| Education |  |  |
| >Level A | 1.00 |  |
| Level O or equivalent | 1.74 | 1.21 – 2.52 |
| <Level O or equivalent | 1.92 | 1.37 – 2.71 |
| Osteoarthritis, (yes) | 3.31 | 2.54 – 4.31 |
| Falls, (yes) | 1.34 | 1.02 – 1.76 |
| Use of corticoids, (yes) | 2.34 | 1.73 – 3.17 |

OR = Odds Ratio; CI = Confidence Interval; BMI = Body Mass Index.

**Supplementary Table 5** – Adjusted logistic regression models for chance of dynapenia and variation in odds ratio (*OR*) according to different groups of diabetes classification in men (n=2,406) and women (n=2,884) aged ≥50 years old, ELSA (2012-2013).

|  | Men^1^ | | Women^2^ | |
| --- | --- | --- | --- | --- |
| Models | **Dynapenia**  ***OR* (95%CI)**  **n=2,406** | **Percentage variation compared to Model 1(%)** | **Dynapenia**  ***OR* (95%CI)**  **n=2,884** | **Percentage variation compared to Model 1(%)** |
| Model 5 |  |  |  |  |
| ND | 1.00 |  | 1.00 |  |
| CD+UDD | 1.34(0.82-2.19) | -19.8 | 0.83(0.54-1.82) | -25.2 |
| UCD | 2.39(1.37-4.17) | +0.84 | 1.68(1.01-2.79) | +0.6 |
| Model 6 |  |  |  |  |
| ND | 1.00 |  | 1.00 |  |
| CD | 1.67(0.95-2.93) | 0 | 1.11(0.68-1.83) | 0 |
| UCD+UDD | 1.69(1.04-2.76) | -28.7 | 1.05(0.68-1.63) | -37.1 |
| Model 7 |  |  |  |  |
| ND | 1.00 |  | 1.00 |  |
| UDD+CD+UCD | 1.68(1.13-2.50) | -29.1 | 1.08(0.77-1.52) | -35.3 |

CI: confidence interval; ND: non-diabetic; UDD: undiagnosed diabetic; CD: controlled diabetic; UCD: uncontrolled diabetic. ^1^Models for men were controlled by age, marital status, family wealth, lung disease, osteoarthritis, osteoporosis, depression, falls, and BMI. ^2^Models for women were controlled by age, marital status, education level, osteoarthritis, falls, and use of corticoids.

**Supplementary Table 6** – Final linear regression models for association between HbA1c and GS in men and women ≥50 years old, ELSA (2012-2013).

| **Final linear regression model** | | | |
| --- | --- | --- | --- |
| **Variables** | **β coefficient** | | **(95%CI)** |
| **Men (n=2,406)** |  | | |
| Age, (years) |  |  | |
| 50-59 | 1.00 |  | |
| 60-69 | -2.87 | - 3.75 to -1.99 | |
| 70-79 | -7.82 | - 8.81 to -6.82 | |
| 80-89 | -13.1 | - 14.5 to -11.7 | |
| 90 or older | -19.5 | - 23.8 to -15.2 | |
| Marital status, (with conjugal life) | -1.12 | -1.90 to -0.34 | |
| Education |  |  | |
| > Level A | 1.00 |  | |
| Level O or equivalent | -1.17 | -2.00 to -0.34 | |
| < Level O or equivalent | -1.59 | -2.38 to -0.80 | |
| HbA1c% |  |  | |
| < 6.5 | 1.00 |  | |
| ≥ 6.5 to <7.0 | -1.62 | -3.19 to -0.04 | |
| ≥ 7.0 to <8.0 | -3.73 | -5.56 to -1.90 | |
| ≥ 8.0 | -2.05 | -4.06 to -0.04 | |
| Stroke, (yes) | -1.25 | - 2.99 to 0.49 | |
| Osteoporosis, (yes) | -2.43 | - 4.39 to -0.46 | |
| Osteoarthritis, (yes) | -1.93 | - 2.66 to -1.19 | |
| Cancer, (yes) | -1.85 | - 3.31 to -0.39 | |
| Depression, (yes) | -3.00 | -4.21 to -1.78 | |
| Dementia, (yes) | 1.61 | -1.90 to 5.13 | |
| Falls, (yes) | -1.91 | -2.82 to -1.00 | |
| BMI – kg/m^2^ |  |  | |
| Ideal | 1.00 |  | |
| Underweight | -4.88 | -10.3 to 0.56 | |
| Overweight | 1.69 | 0.86 to 2.52 | |
| Obesity | 2.89 | 1.93 to 3.86 | |
| **Women (n=2,884)** | **β coefficient** | **(95%CI)** | |
| Age, (years) |  |  | |
| 50-59 | 1.00 |  | |
| 60-69 | -1.16 | -1.73 to -0.59 | |
| 70-79 | -3.39 | -4.03 to -2.75 | |
| 80-89 | -7.20 | -8.11 to -6.28 | |
| 90 or older | -9.59 | -11.98 to -7.20 | |
| Education |  |  | |
| > Level A | 1.00 |  | |
| Level O or equivalent | -1.20 | -1.76 to -0.64 | |
| < Level O or equivalent | -1.64 | -2.18 to -1.10 | |
| HbA1c% |  |  | |
| < 6.5 | 1.00 |  | |
| ≥ 6.5 to <7.0 | 0.15 | -0.91 to 1.21 | |
| ≥ 7.0 to <8.0 | -0.51 | -1.88 to 0.86 | |
| ≥ 8.0 | -1.77 | -3.21 to -0.33 | |
| Stroke, (yes) | -1.91 | -3.13 to -0.69 | |
| Osteoporosis, (yes) | -1.17 | -1.83 to -0.51 | |
| Osteoarthritis, (yes) | -2.13 | -2.57 to -1.69 | |
| Depression, (yes) | -1.48 | -2.11 to -0.85 | |
| Dementia, (yes) | -5.08 | -8.40 to -1.76 | |
| Falls, (yes) | -0.59 | -1.12 to -0.05 | |
| Use of corticoids, (yes) | -1.85 | -2.51 to -1.20 | |
| Abdominal obesity, (yes) | 0.49 | 0.05 to 0.94 | |

GS = Grip Strength; CI = Confidence Interval; BMI = Body Mass Index.
